# Bladder Worms

**Published:** 1884-07-20

**Authors:** E. H. M. Parham

**Affiliations:** Arkansas


### BLADDER WORMS.

By E. H. M. PARRHAM, M.D., OF ARKANSAS.

The subject of this article is of small size, weighing about a hundred and twenty pounds, usually healthy, until last Christmas, when he felt slight pain and soreness in the region of his kidneys (his occupation is teamster at a saw mill), which continued to grow worse, attended with considerable weakness of the inferior extremities, and about two or three weeks ago he applied for medical advice. His personal appearance did not then, nor does it now, indicate disease of any kind, and he is still attending to his occupation. He was directed to take half dram doses of compound fluid extract of buchu. Very soon after he commenced using the buchu he discharged in his urine several living worms, and continues to discharge them occasionally. To-day he passed seven, which I have now before me: they are from one-half to an inch and a quarter long, with dark colored spots or bands on them; size, about twice as large as No. 8 spool thread; general color, white. I have written this article hoping that some experienced member of the medical profession, through your valuable journal, may favor me with a treatment that would prove successful in the management of this case, which is entirely new to me.

[REMARKS BY THE EDITOR.—The writer has requested our views of the above singular case. We have not, in our experience, seen any worms answering to those which Doctor P—— describes. In the books there are only a few varieties of bladder parasites mentioned. Of these, a variety styled "*Dactylius Aculeatus*" answers more nearly the description given in the above case than any other we have seen. Dunglison thus defines this species: "*A worm of a light color, annulated, cylindrical, but tapering slightly toward both extremities, from two-fifths to four-fifths of an inch long.*"

In case of a fistulous opening between the rectum and bladder, *ascarides* might find entrance into the cavity of the latter; but the worm mentioned does not answer in all respects to that of ascarides. Nor does the writer mention any symptom indicating such a condition.

Whatever be the variety of bladder worm that may exist, the principles of treatment are the same.

We would suggest, in a case like the above, a dose of calomel, followed with small, repeated doses of santonine, alternated with the oil of turpentine, conjoined with such diuretics as would direct the vermifuge to the kidneys. A combination of balsam copaiba with turpentine might meet this requirement. The remedy would be more likely to pass off by the kidneys if the bowels were somewhat restrained; not with opium, which suppresses the urinary secretion, but with burnt brandy, ginger, camphor, etc.

The parasites being removed, tonics and bracing treatment should be used to build up the general health. W.

